# Jumping without Using Legs: The Jump of the Click-Beetles (*Elateridae*) Is Morphologically Constrained

**DOI:** 10.1371/journal.pone.0020871

**Published:** 2011-06-16

**Authors:** Gal Ribak, Daniel Weihs

**Affiliations:** Technion Autonomous System Program and the Faculty of Aerospace Engineering, Technion, Haifa, Israel; University of Oxford, United Kingdom

## Abstract

To return to their feet, inverted click-beetles (Elateridae) jump without using their legs. When a beetle is resting on its dorsal side, a hinge mechanism is locked to store elastic energy in the body and releases it abruptly to launch the beetle into the air. While the functional morphology of the jumping mechanism is well known, the level of control that the beetle has over this jumping technique and the mechanical constraints governing the jumps are not entirely clear. Here we show that while body rotations in air are highly variable, the jumps are morphologically constrained to a constant “takeoff” angle (79.9°±1.56°, n = 9 beetles) that directs 98% of the jumping force vertically against gravity. A physical-mathematical model of the jumping action, combined with measurements from live beetle, imply that the beetle may control the speed at takeoff but not the jumping angle. In addition, the model shows that very subtle changes in the exact point of contact with the ground can explain the vigorous rotations of the body seen while the beetle is airborne. These findings suggest that the evolution of this unique non-legged jumping mechanism resulted in a jumping technique that is capable of launching the body high into the air but it is too constrained and unstable to allow control of body orientation at landing.

## Introduction

Click-beetles (Elateridae) are among the record holding insects that apply powerful catapult leverages to jump to heights (or distances) of many body lengths [1]–[5]. Interestingly, they do this without using their legs. A click-beetle finding itself on its back with nothing to grip nearby, will right itself by rapidly flexing the body. The flexion launches the beetle high into the air at accelerations of up to 380 times gravity [5]. While in air the body can rotate vigorously [5]–[6]. However, the probability of the beetle for landing on its feet is not different than random chance [5], [7].

**Figure 1:**
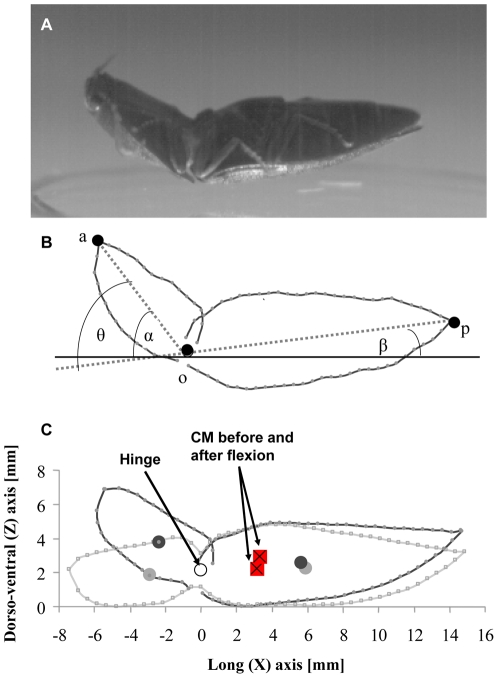
Jumping click beetles. A) a beetle just after leaving the ground showing the flexed body. B) a modeled beetle seen from the side. ‘a’, and ‘p’ denote the tip of the head and abdomen respectively. ‘o’ denotes the hinge. θ is the flexion angle, α and β are the angles between the long axes of the subunits and the horizontal. C) a model beetle in the pre-jump position (grey) and after flexing the body (black). Circles denote the center-of-mass of the subunits and red rectangles with an x denote the center-of-mass (CM) of the entire body in the pre-jump posture and after flexing.

Evans [5]–[6] described the jumping of click-beetles in depth. He showed how the hinge that functionally divides the body into two subunits is locked by a cuticular peg while a large longitudinal muscle connecting the two subunits contracts to store elastic energy. When the peg slides and unlocks the hinge the stored energy is abruptly released, flexing the body ventrally (Fig. 1) within less than 1 ms. The beetles he studied (*Athous haemorrhoidalis*, with body length 10–12 mm) jumped to a height of up to 30 cm (i.e. >25 body lengths) and performed up to six somersaults in the air before landing [5]. However, to flip back to its feet a click-beetle needs only to elevate its body by one body length and perform a half of a full revolution. Thus, the jumps of the click-beetles grossly exceed the minimal requirements for righting. This excess power output and the ∼50% probability of landing back on the feet suggest that the beetles are incapable of evaluating the forces and torques needed to flip over. Why this particular form of jumping can not be controlled more precisely is not clear.

Intrigued by the evolution of a unique righting mechanism that seems to be deprived of means to control the orientation of the body during the jump and landing, we investigated how a fixed elastic action results in different body orientation at landing every time. We analyzed the jumps of *Lanelater judaicus* from movies showing the trajectory of the beetles in air. The kinematics data and beetle morphology were used to construct a biomechanical model of the jumping mechanism. The model was then used to evaluate the jumping constraints from the mechanical relation between the flexion dynamics of the body and the resulting aerial maneuver.

## Materials and Methods

All animal work was conducted according to the national guidelines. *Lanelater judaicus* (Fig. 1A) were collected in Northern Israel (N32°42.407, E35°13.782) during the summer. A collection permit for these beetles in that site was not required by law, but the work was approved by the Permit department of the Israel Nature and Park Authority. Experiments were performed within a week of collection. The beetles used in the experiments (n = 9) had a mean body length of 20.3 mm (s.d. = 2.3) and body mass of 0.20 g (s.d. = 0.05).

### Jump kinematics

**Figure 2:**
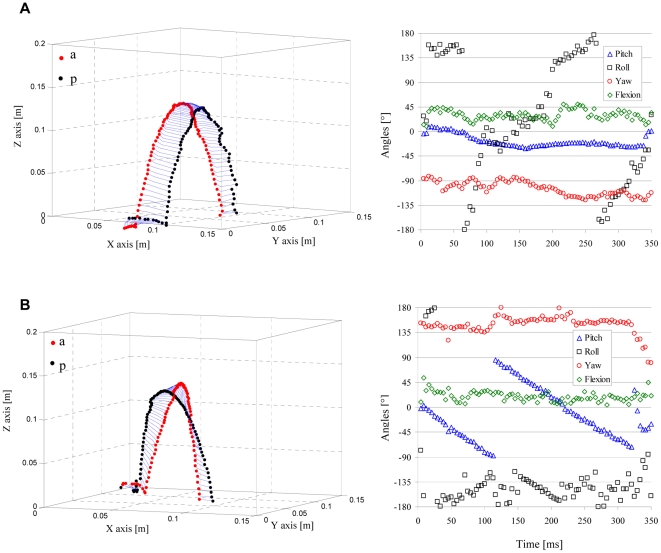
3D kinematics of the aerial maneuver during the jump of the click-beetle. Top and lower panels are two examples of data extracted from movies. Figures on the left show the ballistic trajectory through the 3D positions and orientation of the tip of the head (a, red), tip of the abdomen (p, black) and the lines connecting them through the hinge (blue) in each video frame (See Fig. 1B). Figures on the right show the instantaneous flexion of the body (green), yaw (red), pitch (blue) and roll (black) angles as a function of time for the jumps on the left. A is an example for a jump in which the beetle mostly rolled and B shows a jump where the beetle somersaulted (pitch) in the air.

The aerial maneuver of 9 individuals of *L. judaicus* were filmed either at 240 frames s^−1^ using one digital camera (Epix Silicon 642M) and two mirrors (26×30 cm each) or later at 250 frames s^−1^ using two Photron SA3 cameras. The mirrors were placed perpendicular to each other and angled at 45° to the line of sight of the camera. When a beetle was positioned between the mirrors, it was visible to the camera from the front directly and from two sides through the mirrors. To position the beetle on its back between the mirrors with minimal handling interference we used a piece of smooth cellulose paper placed in the path of a walking beetle. Once the beetle walked over the paper, the paper was gently raised and made vertical above the desired location. The beetle lost foot-hold on the slippery surface and slid off the paper onto its back. Typically, the beetle first attempted to find a foot hold that could aid in righting by swinging all legs through the air. After several futile trials they tucked their appendages close to the body, assumed the pre-jump posture [5] (Fig. 1C) and jumped. The different camera views were spatially calibrated using DLT coefficients obtained from a rectangular object with known dimensions [8]. The calibration of the cameras allowed to extract the 3D translations and rotations of the beetles in air from the video sequences (Fig. 2, see Text S1).

### Body geometry

The shape and dimensions of the body extracted from orthogonal images of 4 dead beetles were used to construct geometrically simplified models of the body (Fig. 1, Text S1 and Figure S1). We treated the body as two rigid subunits rotating about a frictionless hinge [6]. Assuming uniform density of body tissues, we calculated for each beetle the position of the center of mass for the entire body and for each of the two subunits separately. The reliability of this estimation was qualitatively verified by balancing the dead beetles on a razor blade. In the pre-jump posture the observed longitudinal position of the center of mass roughly coincided with the point of maximum thickness of the posterior subunit, in agreement with estimates derived from the models (Fig. 1C). On the transverse axis, the center of mass was assumed to be located on the line of bilateral symmetry. The models were used to calculate the mass moment-of-inertia (for rotation about the transverse axis = pitch) of the two subunits about their center of mass and about the hinge (Text S1, Figure S1).

### Biomechanical model

Manipulation of the dead beetles revealed that rotation of the subunits about the hinge was constrained by the morphology of the rigid exoskeleton. The flexion angle of the body (θ), measured between the long axes of the subunits (Fig. 1B), was approximately 0° at the pre-jump posture and reached 55° at the end of body flexion. The angle θ is a sum of angles α and β representing the angular displacement, relative to the horizontal, of the long axes of the anterior and posterior subunits, respectively (Fig. 1B). When the beetle starts the jump the elastic energy stored in the musculature and cuticle converts to an intrinsic couple (*M*) that rotates the subunits towards each other. The moment flexing the body was assumed not to vary over the short time of the flexion (dM/dt = 0). Consequently, the angular acceleration of each subunit was taken as constant and each subunit rotated according to [9]:
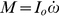
(1)

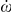
 is the angular acceleration in the sagittal plane and *I_o_* is the moment of inertia of the subunit about the hinge.

Both subunits are rotated by the same moment (but in opposite directions) so that:
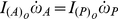
(2)the subscripts _A_ and _P_ denote the anterior and posterior subunits respectively. After rearranging:
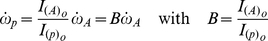
(3)We find that the angular accelerations of the two subunits are related through a morphological property, *B*, which is a constant - the ratio of the moment-of-inertia of the two subunits. Writing the constant acceleration equations for α and β for the duration of the flexion (*t_f_*):
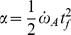
(4a)

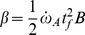
(4b)we obtain:
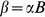
(5)recalling that 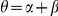:
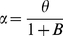
(6a)

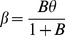
(6b)Thus, each θ will have a single pair of α and β values regardless of the angular accelerations or time to complete the flexion. In fact, to find α and β for a given value of θ one needs to know only the ratio of the moment of inertia of the two subunits, *B* (Equations 3, 6). The four modeled beetles were used to calculate an estimate for B (Text S1 and equation 3) and from it the angles α and β. Once these angles were found we were able to calculate the forces and torques that power the jump. The calculation is briefly explained below. The full description appears in the supporting information section (section C of Text S1).

When the body, resting against the ground, changes from the pre-jump to the flexed posture the center of mass is accelerated upwards launching the beetle into the air [5]. Once B was known and α and β found (Eq. 6), we rotated the two subunits in our modeled beetles about the hinge, to the flexed position to find the new position of the center of mass (Fig. 1C). From the shift in the center of mass and the speed at takeoff we were able to calculate the force that launches the body into the air (Text S1). Just before leaving the ground, the beetle is resting on the curved anterior edge of the elytra with most of the body in air (Fig. 1). The angular speed that rotates the body in the air is added to the beetle at takeoff when the point of contact with the ground is not positioned on the line of action of the launching force. Thus, small changes in the position of the point of contact with the ground will alter the torque generated at take-off. Using the geometry of the beetle and the launching force we simulated the moment and resulting angular velocities that will ensue for different points of contact with the ground (Text S1).

## Results

**Figure 3:**
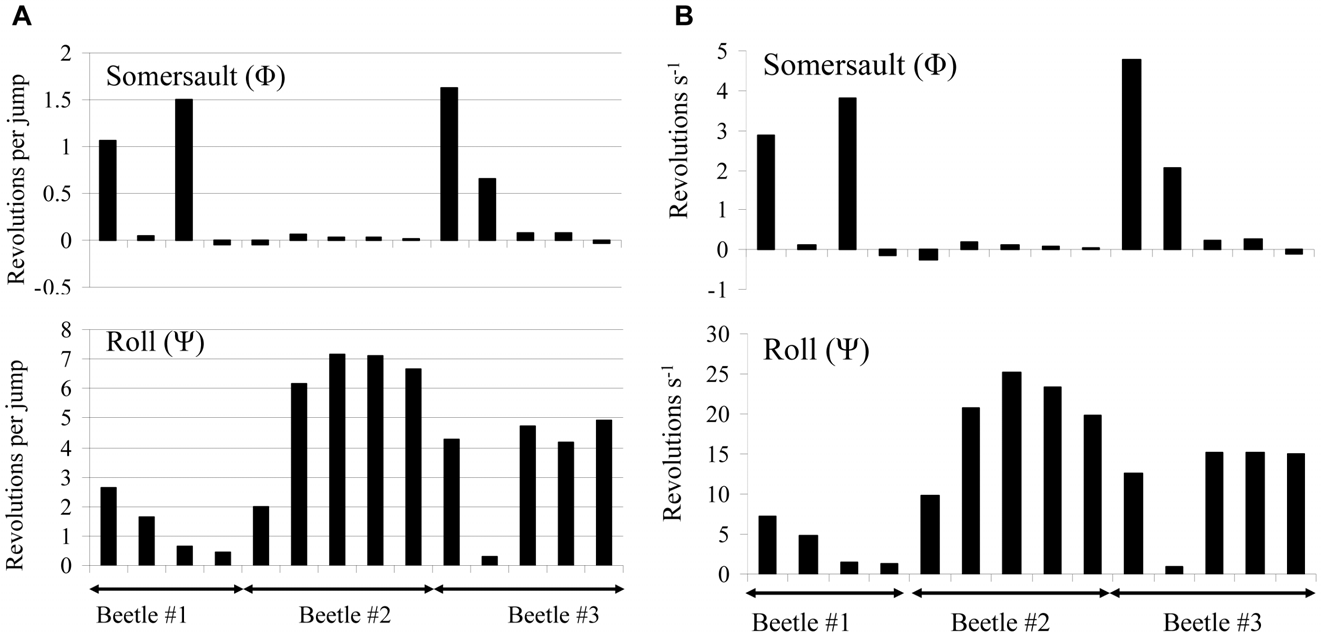
Body rotations in the air. The mean rotation rate observed in 14 jumps made by three different beetles. Left figures (A) show the number of revolutions per jump and the same data is represented on the left (B) as revolutions per second. The upper and lower figures refer to somersaults and rolls respectively. Each column represents a single jump.

**Figure 4:**
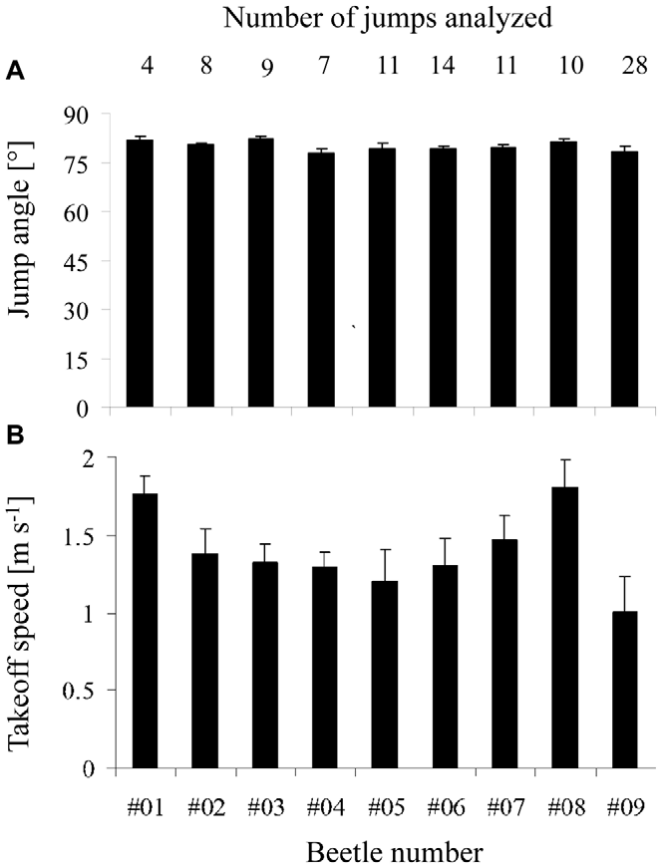
Observed jump angles (A) and takeoff speeds (B). Each column represents the mean of one out of 9 beetles. The error bars is 1 s.d. The number of jumps observed per beetle is denoted at the top of the figure.

The live beetles were observed somersaulting and/or rolling in the air. Figure 2 shows two examples of the 3D kinemtaics extracted from video sequences. In the first (Fig. 2A) the beetle rolls while in the air while in the second (Fig. 2B) the beetle somersaults (Fig. 2B). Figure 3 gives the mean rotation rates of the beetles in the air in 14 jumps by three different beetles. Although the beetles were jumping unobstructed from the same, flat and rigid surface, the rotations varied in angular speed between individuals as well as between the different jumps of the same beetle (Fig. 3). Furthermore, the same beetle could be seen rolling in one jump and pitching (somersaulting) in the next. Figure 4 shows that the speed at takeoff was variable, but the jump angle was remarkably conserved. The jump angles observed in 102 jumps by 9 beetles were between 75.0°, and 83.4°. Calculating the mean jump angles for each individual (79.9°, s.d. = 1.56°, n = 9) showed that the s.d. in jump angle for the beetle with the highest observed variation (beetle#09, Fig. 4) was only 1.8°.

Substituting θ = 55° and the mean value for *B* calculated from our modeled beetles (*B* = 0.124, s.d. = 0.006, n = 4) in equation 6 yields α = 48.93° and β = 6.07°. Using these angles to rotate the two subunits about the hinge in the models, we found that the translation of the center of mass (*d_cm_*), as the body changed from the pre-jump to the flexed posture, was *d_cm_* = 0.7±0.06 mm. The translation was towards the posterior and up (ventral side) at an angle of 77.6±1.33° (mean ± s.d. of n = 4) to the horizontal. This angle constitutes the jump angle predicted by the models. The predicted angle (77.6°) was only 2.3° smaller than the mean angle observed in live beetles (Fig. 4).

**Figure 5:**
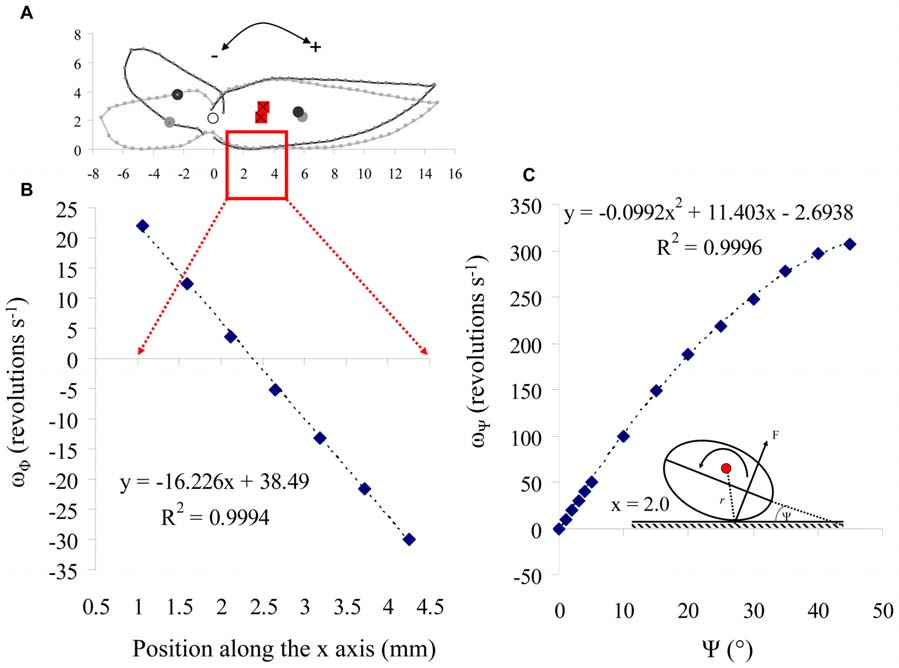
Model simulation of the angular velocity. A) The modeled beetle in figure 1C showing that the point of contact with the ground should be within the red rectangle. The x axis of the rectangle is magnified in B. B) The model calculation for angular speed of somersaulting (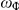) as a function of the point of contact with the ground along the x axis. C) Model calculation of angular speed for rolling in the air (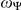) as a function of roll angle ψ of the body in the pre-jump posture. The longitudinal position of the point of contact with the ground is assumed to be x = 2 mm posterior to the hinge. The insert in C shows the beetle in cross-section, defining ψ and the moment arm. Red circle denote the center-of-mass.

The simulations of angular speed at takeoff (Fig. 5) showed that from the point of zero torque (2.4 mm posterior to the hinge) each longitudinal shift of the point of contact with the ground by 0.5 mm (2.5% of body length) towards the anterior of the body linearly increased the rate of somersaulting by 8.1 revolutions s^−1^. Simulations of roll showed that rotation of the body (in the pre-jump posture) about the long axis by 1° (from 1° to 2°) increased the speed of roll from 8.6 to 19.91 revolutions s^−1^. The variability of observed rotation rates in live beetles (Fig. 3) is within similar ranges.

**Figure 6:**
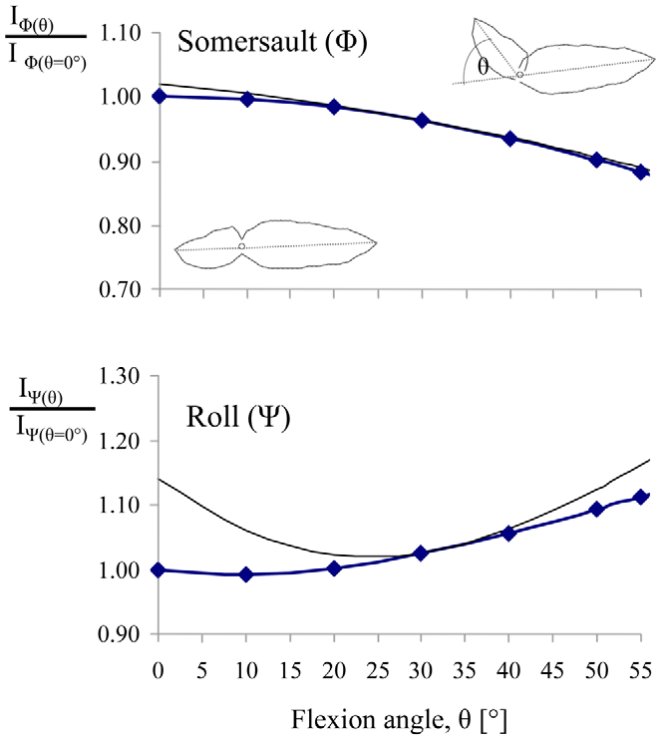
Proportional change in moment-of-inertia for pitch (I_Φ_) and roll (I_Ψ_) as a function of θ. The moment of inertia of the body for a given flexion angle (0<θ<55°) is divided by the moment of inertia of the beetle at the pre-jump posture (θ = 0°). Two alternative calculations are shown. Data points and the thick lines represent calculated estimates using the instantaneous center of mass. The thin lines are the same estimates calculated assuming the center of rotation stays at the initial position of the center of mass as the beetle left the ground (i.e. θ = 55°).

Adjusting the flexion angle of the body after takeoff has limited effect on the moment of inertia of the entire body about the center of mass. For both pitch and roll the changes to the estimated moment of inertia is only 10% when the flexion angle is changed between θ = 0° to θ = 55° (Fig. 6).

## Discussion

Our analysis aimed to evaluate the degree of control that the beetles might have over jumping. Such control can be divided to controlling jump trajectory and to controlling the orientation of the body in air. According to the model, when the beetle flex to power the jump, the angular displacements of the two subunits of the body (α, and β, Fig. 1B) are independent of flexion duration (Eq. 6). Since the combination of these angles determine the displacement of the center of mass (*d_cm_*) during the flexion action, we obtain that the size of *d_cm_* and its angle relative to the horizontal are set to a constant by the rigid morphology the beetle. The direction of the force that acts on the center of mass and launches the beetle into the air, has the same direction as *d_cm_*. Hence, the beetles are constrained to a constant jump angle and this prediction by the model is strongly supported by the observed jump angles in live beetles (Fig. 4). The jump angle (79.9°) is almost vertical directing more than 98% of the jumping force vertically against gravity.

In contrast to the fixed direction of the launching force, the magnitude of this force need not be fixed by the rigid morphology. The magnitude depends not only on *d_cm_* but also on the time it takes the body to flex (Text S1). Within the time it takes the body to flex, the center of mass is accelerated from its pre-jump (resting) position to the flexed position (Fig. 1C). The shorter the duration of flexion, the higher the acceleration that is responsible for launching the beetle into the air. The duration of flexing the body by 55° in itself depends on the muscle contraction that stores the elastic energy for flexion in the pre-jump posture (while the hinge is locked). A weaker contraction will store less elastic energy resulting in the buildup of less torque about the hinge. Upon unlocking the hinge the two subunits will rotate at lower angular accelerations taking longer to complete the flexion motion. The center of mass will take longer to move the same distance (*d_cm_*), resulting in reduced acceleration and takeoff speed. Hence, the beetles may control the speed at takeoff by adjusting muscle contraction in the pre-jump posture. Indeed the takeoff speed observed in live beetles (Fig. 3) is much more variable than jump angle.

However, the ability to only adjust takeoff speed leaves the beetle with limited control over the jump trajectory. The constant jump angle implies that a ballistic trajectory has only one horizontal distance value for any jump height (vertical distance) value. Adjusting takeoff speed to increase jump height always leads to an increase in the horizontal distance as well.

Regarding the control of rotations of the body while airborne, not only are the rotations inaccurate and often excessive for righting purposes, it also seems that each jump has its own combination of somersaults and/or rolls (Fig. 3). Spring-tails (Collembola) also perform non-legged jumping and they are reported to tumble while in the air [10]–[11]. Few similar reports on tumbling exist also for insects jumping by rapid release of elastic energy in their legs [12]–[13]. This inability to control body orientation in the air has been attributed to ‘startle’ jumps executed for escaping predators [10]–[11] but can occur also in targeted jumps of some insects that do not stabilize body orientation in the air with their wings [12]–[13]. In the case of the click-beetle, the high variability in body rotations seen in live beetles, can be explained by our model. Prior to leaving the ground the beetle is resting on the curved anterior edge of the elytra. Calculations show that when balanced on the curved surface, subtle changes in the exact point of contact with the flat ground can have a dramatic effect on the speed and type of rotations of the body in air (Fig. 5).

Since the beetles often leave the ground with very high angular velocity, the conservation of angular momentum requires that in the absence of significant air resistance they will continue to rotate while airborne. However after leaving the ground the angular speed of rotation can be somewhat altered without changing the angular momentum by changing the moment of inertia of the body. Human athletes and falling cats adjust body configuration in the air to adjust the moment of inertia thus controlling the rotation of the body to some extent [14]–[15]. We used our beetle model to check how changes in the flexion angle of the body, while in the air, alters the moment of inertia for pitch and roll (Text S1). Figure 6 shows that in both cases changing the flexion angle of the body between 0 and 55° results in no more than a 10% change in the moment of inertia of the entire body for pitching (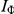) and rolling (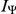). Such a change will result in a maximum change of ±10% in the speed of rotation. It is not clear that the beetle make use of this option, but with rotations rates of 3–5 somersaults s^−1^ (or 20 rolls s^−1^), the 10% adjustment on rotation rate is probably insufficient to slow the rotation of the beetle enough to allow controlled landing in the right orientation.

**Figure 7:**
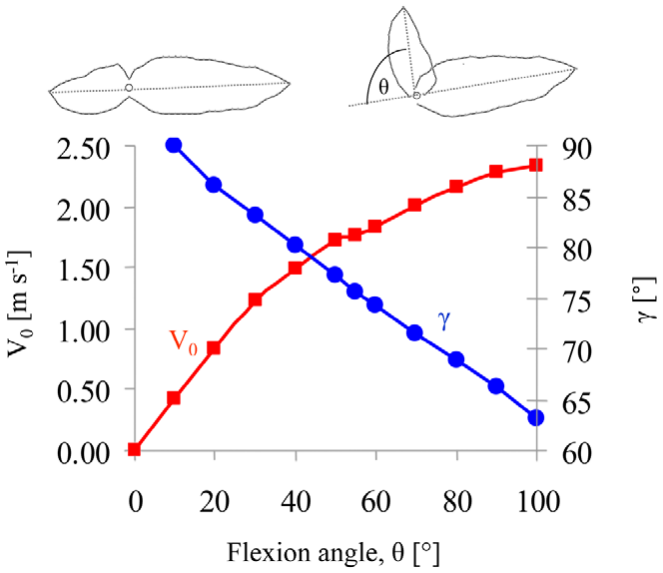
Simulated takeoff speed (V_0_) and jump angles (γ) for a wide range of θ values.

Click-beetles will jump as a defensive response as well as for righting [5]. However when resting on their dorsal side they will right themselves by jumping even in the absence of a threat. Since the jump angle is dictated by the flexion angle we explored the evolutionary significance of this particular flexion angle compared to hypothetical alternatives. We used the model to simulate hypothetical scenarios changing the flexion angle while keeping the angular acceleration of the subunits the same. Calculating from the model the speed at takeoff and jumping angle as a function of flexion angles we found that there is a tradeoff between takeoff speed and a vertical jumping angle (Fig. 7) so that θ = 55° provides relatively high takeoff speeds while keeping the jump angle relatively vertical. We therefore suggest that the jumps of click beetles evolved primarily as a mechanism for vertical jumping. This supports the idea that the jumps are an adaptation for righting as opposed to an adaptation as an escape response. The logic is that jumps at shallow angles of 30° to 45° would have been far more effective in distancing the beetle from its attacker. It also seems unlikely that an escape mechanism would evolve based on jumping in a highly predictable angle each time. Keeping all jumps vertical may ensure sufficient height (and time in the air for rotating) when jumping from soft substrates such as foliage or loose soil. With no means of controlling body rotations or altering jump angle, the righting behavior of click-beetles seems to resolve to jumping as high as possible, relying on random chance for landing back on their feet.

## Supporting Information

Text S1 A) Analysis of high-speed movies. B) Estimating the center of mass and mass moment of inertia from modeled beetles. C) Estimating the force, torques and angular velocities of body rotation developed by the jumping action.
(DOC)

Figure S1 Example of body contours used to model the beetle geometry. A) Planform view, the body is modeled as two halves of ellipses with a common minor axis at the location of the hinge (x = 0, y = 0). B) Side view based on images of the body in the pre-jump posture. The position of the Center of mass (cm) is denoted by the encircled×symbol. C) Notations of diameters and axes for each volume element (see also Text S1).
(DOC)
