# Supplementary material for: Jumping without Using Legs: The Jump of the Click-Beetles (Elateridae) Is Morphologically Constrained
Source: PLoS One. 2011 Jun 16;6(6):e20871. doi: 10.1371/journal.pone.0020871 (PMC3116849; doi:10.1371/journal.pone.0020871)
Supplement: Text S1 — A) Analysis of high-speed movies. B) Estimating the center of mass and mass moment of inertia from modeled beetles. C) Estimating the force, torques and angular velocities of body rotation developed by the jumping action. (DOC) [file pone.0020871.s001.doc]

**Text S1- Supporting Information**

**A. Analysis of high-speed movies**

We used the position of the hinge to represent the position of the beetle in the video frames. For each jump, we measured the maximal vertical displacement of the beetle (jump height = h_max_) and the total horizontal distance the beetle traversed in the air during the jump (R). The takeoff speed (V_0_) and jumping angle (γ, measured from the horizontal plane) in which the beetle left the ground were found using the equations of projectile ballistics (neglecting the viscous drag on the beetle):

$h_{\max}=\frac{V_{0}^{2}\sin^{2}\gamma}{2g}$ (A1a)

$R=\frac{V_{0}^{2}\sin2\gamma}{g}$ (A1b)

where *g* is the acceleration due to gravity (9.8 ms^-2^).

Rearranging Eq. A1a gives:

$V_{0}=\sqrt{\frac{2gh_{\max}}{\sin^{2}\gamma}}$ (A2)

and substituting Eq. A2 in Eq. A1b gives:

$R=\frac{2gh_{\max}}{\sin^{2}\gamma}\frac{\sin2\gamma}{g}=\frac{2h_{\max}2\sin\gamma\cos\gamma}{\sin^{2}\gamma}=4h_{\max}\tan^{-1}\gamma$ (A3a)

$\Rightarrow\tan^{-1}\gamma=\frac{R}{4h_{\max}}$ (A3b)

The vertical acceleration of the beetle in air, as measured from the second time derivative of the instantaneous position of the beetles in the movies (9.77 ± 0.015 m s^-2^, n=3 beetles), was very close to standard gravitational acceleration (9.8 ms^-2^) corroborating the assumption that air resistance is negligible for the sake of this analysis.

To extract the rotations of the beetle in air we took advantage of the fact that the body of the beetle is comprised of two rigid subunits connected at the transverse hinge. The prothorax and head form the anterior subunit and the mesothorax, metathorax and abdomen form the posterior subunit (Fig. 1). We randomly selected 14 jumps from 3 different beetles and in these movies we digitized three points on the beetle in each video frame showing the beetle in air (see Figure 1B): the anterior tip of the body (a), the posterior tip (p) and the hinge (o). The hinge only allows rotation of the body's subunits in respect to each other in a plane perpendicular to the hinge. Furthermore, the rotation of the subunits in that plane is limited to rotations towards the ventral side of the beetle. Thus, from the flexion of the body in the films, it was possible to define the sagittal plane of the beetle and to distinguish the dorsal side of the beetle from the ventral side. A morphological coordinate system (x,y,z) fixed in the beetle was defined and the direction cosines of these morphological axes were found in the lab based coordinate system (in which Z was vertical, X and Y were horizontal). To find the morphological axes, points "o", "a" and "p" were used to find the vectors **oa** and **op** that both lie in the sagittal plane of the beetle (Fig 1B). The vector normal to this plane (**y** = **oa**×**op)** was taken as the transverse axis of the body. The vector **po** was taken as the long axis of the body (**x,** positive towards the head). The dorso-ventral axis of the body was found from **z = po** × **y** (positive towards the dorsal side). The direction cosines of **x**, **y** and **z** in the lab based coordinate system was used to construct a rotation matrix from which we extracted the instantaneous Euler angles (yaw, pitch and roll) at each time step (movie frame) of the aerial maneuver (Fig. 2). Since yaw is irrelevant to flipping the beetle between the dorsal and ventral side our analysis focused on pitch and roll. For each jump we measured the total angular displacement for each Euler angle and divided it by the duration of the aerial phase to obtain mean angular velocities for pitch and roll.

**B. Estimating the center of mass and mass moment of inertia from modeled beetles**

4 beetles were measured for mass, body length, maximum width (left-right) and maximum dorso-ventral thickness. The planform view of the beetle (in the xy plane, i.e. coronal plane, see Figure S1) was assumed to have the shape of two half ellipses with a common center at the hinge and a common minor axis (maximum width of the body) but having different major semi-axes. For the Sagittal plane, the contour of the dorsal and ventral sides of the body in the pre-jump posture were digitized from images at 43 equally spaced points along the length of the body. The body contour data was used to divide the body into 42 volume elements ('slices') along the length of the beetle. We assumed that each slice is an elliptical cross-section and found the position of the centroid of the slice and the average diameters of the ellipse forming the perimeter of the slice. From the diameters and slice thickness we estimated slice volume from the formula for an elliptical cylinder (V_n_= πy_n_z_n_x_n_/4, where V_n_ is the volume of the n-th slice and y_n_ and z_n_ are the average diameters of slice n along the y and z axes respectively; x_n_ is the thickness of the slice, see Figure S1). The mass of the beetle was then divided by the summed volume from all slices to give the average density of the body. Multiplying the volume of each slice by the density gave an estimate of the mass of each slice. We then assumed that the center of mass of each slice is located at the centroid and calculated a mass moment for the slice by multiplying the mass of the slice by its position along each axis of the body. By summing up all the resulting mass moments per axis and dividing by the total mass of the beetle we obtained the position of the center of mass of the beetle. The calculation was also repeated for each subunit separately to find their center of mass as well. In the next step we calculated the mass moment of inertia of each subunit about its center of mass. For this we first calculated the moment of inertia of each slice for rotation about the x, y, and z axes passing through the centroid of the slice (Figure S1). For an elliptical cylinder the equations are:

$Ixx_{(n)}=\frac{m_{n}(y_{n}^{2}+z_{n}^{2})}{16}$ (rotation about the x axis) (B1a)

$Iyy_{(n)}=\frac{m_{n}z_{n}^{2}}{16}+\frac{m_{n}x_{n}^{2}}{12}$ (rotation about the y axis) (B1b)

$Izz_{(n)}=\frac{m_{n}y_{n}^{2}}{16}+\frac{m_{n}x_{n}^{2}}{12}$ (rotation about the z axis) (B1c)

where the subscripts n in the equations above denote that the calculations are for the n-th slice (n=1,2,3…n), *m_n_* denotes the mass of the n-th slice and *y_n_* and *z_n_* are the diameters and x_n_ is the thickness of the n-th slice. We then calculated for each slice the moment of inertia for rotation about the center of mass of the subunit that it belongs to, using the parallel axis (Huygens-Steiner) theorem (*1*):

I_cm(n)_ = I_yy(n)_ + m_n_(r_n_)^2^ (B2)

where r_n_ is the distance from the centroid of the n-th slice to the center of mass of the subunit. The moment of inertia of each subunit about its center of mass was calculated by summing I_cm(n)_ from all slices in the subunit.

The moment of inertia about the hinge was found, for each sub-unit, from the moment of inertia of the subunit about its center of mass using the same parallel axis theorem:

I _o_ = I_cm_ + mr^2^ (B3)

where m is the mass of the subunit and r is the distance between the center of mass of the subunit and the hinge (o).

^1^ S. Timoshenko**,** The Strength of Materials. D. van Nostrand Company, Inc., Toronto (1940)

**C. Estimating the force, torques and angular velocities of body rotation developed by the jumping action**

C1. The force powering the jump

We define *d_cm_* as the translation of the center of mass that results from flexing the body from the pre-jump to the flexed position. The flexion occurs in a time interval (*t_f_*) which is the period it takes the body to performs the flexion through θ =55°. By finding *d_cm_* from the geometry of the beetles, the takeoff speed (*V_0_*) from the trajectory of the beetle in the movies (as described in section A above ) and the fact that the center of mass accelerates from rest, we assumed constant acceleration and calculated the acceleration *a_cm_* and duration (*t_f_*) from:

$a_{cm}=\frac{V_{0}^{2}}{2d_{cm}}$ (C1)

$t_{f}=\frac{V_{0}}{a_{cm}}$ (C2)

The jump is powered by the reaction force from the floor. Multiplying the acceleration found in Eq. C1 with the total mass of the beetle (*m_tot_*) gives the minimal reaction force from the ground that is needed to accelerate the body to the launch speed in *t_f_* seconds:

$F=m_{tot}a_{cm}$ (C3)

Substituting the body dimensions and mean takeoff speeds (V_0_ =1.76, s.d.= 0.122 m s^-1^,n=4) of one beetle (beetle#1 in Fig. 3), we calculated the force powering the jump from the acceleration and mass of the beetle (Eq. C3). From the force we calculated the torques and angular speeds of the body at takeoff (below).

C2. The pitching moment

The last point of contact with the ground is on the anterior end of the posterior subunit (the edge of the elytra). If this point does not lie on the line of action of the ground-reaction force, a torque will ensue, and the beetle will rotate in the sagittal plane (pitch = somersault). The torque responsible for somersaulting (*M_Φ_*) measured at the center of mass of the beetle will depend on the reaction force (Eq. C3) and the distance between the center of mass and the line of action of the force:

$M_{\Phi}=rF\text{sin}\sigma$ (C4)

where *r* here is the distance between the point of contact with the ground and the center of mass and $\sigma$is the angle between *r* and *F*. We calculated *M_Φ_* for a range of possible contact points along the dorsal side of the posterior subunits. For each one of these we found the resulting angular acceleration and angular speed $\omega_{\Phi}$ (for *t_f_*):

(C5a)

(C5b)

where $I_{\Phi}$ is the moment of inertia of the entire beetle for somersaulting about the center of mass. It is found from the moment of inertia of the two subunits as described above in section B but this time for rotation about the center of mass of the beetle:

$I_{\Phi}=I_{\Phi cm(A)}+m_{(A)}r_{A}^{2}+I_{\Phi cm(P)}+m_{(P)}r_{P}^{2}$ (C6)

where the subscripts *A* and *P* denote the anterior and posterior subunits respectively and r represent the distances between the center of mass of each subunit and the center of mass of the entire beetle. The calculation of *I_Φ_* was performed for the body flexed at θ = 55° and also for other flexion angles to show that altering θ in air at a range of 0< θ <55° will increase *I_Φ_* by no more than 10%.

C3. The rolling moment

Due to the bilateral symmetry of the beetle all the forces, translations and rotations described above were in the sagittal plane. Here we introduce a deviation from the 2D analysis which arises when the beetle in the pre-jump posture is slightly rolled to one side and therefore does not rest perfectly on the left-right symmetry line of the body. In such a scenario the point of contact with the ground is lateral to the center of mass and a second torque will form that will roll the beetle about the long (x) axis. To estimate this torque and angular speed we set the longitudinal point of contact with the ground to x= 2mm posterior to the hinge (Figs. 1,5), and simulated the magnitude of the moment arm after rolling the beetle in the pre-jump posture. At the point of contact with the ground the body has an elliptical cross-section. By rotating such an ellipse (with diameters taken from the modeled beetle geometry) by an angle *Ψ* we found the point that was lowest on the perimeter of the ellipse, treated it as the point of contact with the ground and measured the distance (r) to the center of mass of the beetle in the transverse plane. We preformed the analysis for 0< *Ψ* < 45° and calculated the moment *M_Ψ_* as in Eq. C4 (but for the transverse plane). Similarly Eq. C5a and C5b, apply here for roll by substituting the subscript *Φ* with *Ψ*.

(C8)

(C9)

and *I_Ψ_* is the moment of inertia of the beetle for roll about the center of mass of the body.

It is found as in Eq. C6 but using the moment of inertia of the two subunits for roll about their center of mass:

$I_{\Psi}=I_{\Psi cm(A)}+m_{(A)}r_{A}^{2}+I_{\Psi cm(P)}+m_{(P)}r_{P}^{2}$ (C10)

The calculation of *I_Ψ_* was performed for the body flexed at θ = 55° and also for a range of angles to show that altering θ in air at a range of 0< θ <55° will decrease *I_Ψ_* by no more than 10%.
