# Supplementary material for: Jumping without Using Legs: The Jump of the Click-Beetles (Elateridae) Is Morphologically Constrained
Source: PLoS One. 2011 Jun 16;6(6):e20871. doi: 10.1371/journal.pone.0020871 (PMC3116849; doi:10.1371/journal.pone.0020871)
Supplement: Figure S1 — Example of body contours used to model the beetle geometry. A) Planform view, the body is modeled as two halves of ellipses with a common minor axis at the location of the hinge (x = 0, y = 0). B) Side view based on images of the body in the pre-jump posture. The position of the Center of mass (cm) is denoted by the encircled×symbol. C) Notations of diameters and axes for each volume element (see also Text S1). (DOC) [file pone.0020871.s002.doc]

Figure S1

**Figure S1:** Example of body contours used to model the beetle geometry. A) Planform view, the body is modeled as two halves of ellipses with a common minor axis at the location of the hinge (x=0, y=0). B) Side view based on images of the body in the pre-jump posture. The position of the Center of mass (cm) is denoted by the encircled × symbol C) Notations of diameters and axes for each volume element (see also Text S1).
